# Notes on Genito-Urinary Therapeutics

**Published:** 1882-06

**Authors:** 


### NOTES ON GENITO-URINARY THERAPUTICS.

(*Continued from page 231.*)

AGNUS C. RENAL.—When urinating, has pains, sometimes in lower abdomen (Lach., Puls.), sometimes in the kidneys (Berb., Rheum.).

VESICAL.—Pains in bladder (*Ant. t.* Hell., Nux v., Puls., Rhod., Sulph.).

Micturition profuse; gets up twice at night and even once during siesta. (Afternoon while asleep Alum., Caus., Staph. Emission of urine in first sleep Sepia).

URINARY PAINS.—Disagreeable sensation in back part of urethra after micturition.

Burning and pressing in urethra during micturition.

CHARACTER OF URINE.—Urine is red, turbid, with burning and pressure in urethra.

Frequent and darker than usual.

Increased in amount and passed in stronger stream. (Curative effect?)

SEXUAL.—Desire lessened or almost completely lost.

Penis small, flaccid (Calad.) and cold, (Cold: Agar., Cann., Caps., Brom.)

*Penis so relaxed that voluptuous fancies excite no erections.*

Erections without voluptuous desire.

Morning erections but parts are flaccid.

Semen runs out in a stream, without ejaculation. (Involuntary dribbling, Selen; from relaxed penis; Bell., Coral.)

Drawing along spermatic cord. (Berb., Clem., Lact., *Mang.*, *Merc.*, Nitr. ac., Puls., Zinc.)

Testicles feels cold (to others) at night; are swollen, hard and painful (from a suppressed gonorrhœa).

Itching of genitals.

Clinical.—Useful in impotence with gleet, especially in those who have had gonorrhœa often. Gonorrhœal discharge is yellow and purulent (inflammatory symptoms having subsided) there is neither erections nor desire.

Pollutions from irritable weakness with prostratorrhœa.

Concomitants.—Patient is depressed and gloomy; is dissatisfied with himself.

ALOE SOC. Vesical.—At times painful burning in neck of bladder on urinating (Cham., Nux v., Thuja.).

Pain down left ureter.

*Feels as though stool would pass each time he urinates.*

Burning and urging to urinate with general agitation.

Frequent urging to urinate, with no increase in quantity or even in smaller quantity; worse afternoon or night.

Urine passed with difficulty.

Burning when urinating.

Desire so urgent he can hardly retain urine.

After a stool, copious pale urine.

Incontinence of urine (in old men with enlarged prostate).

Character of Urine.—Urine saffron yellow, becoming cloudy; scanty and hot.

Smell putrid or ammoniacal. Bloody.

Sediment yellowish like bran or slimy (slimy, Ars., *Berb.*, Calc., Merc., Natr. c., Natr. m., Puls., Tereb., etc.).

Sexual.—Desire increased; after waking (2 a. m.), after eating, in evening.

Active desire at evening with uncommon thirst.

Desire more active after stool.

Erections in morning (Thuja) and *after* passing water.

Seminal emissions, yet strong desire afterwards.

Semen ejaculated too soon.

Involuntary emissions during siesta (Agar.); toward morning followed by sexual excitement, micturition and stool.

Itching of prepuce after stool.

Testicles cold; right feels cold at night; penis small; scrotum relaxed. (Feels heavy, Am. c., Natr. c., Ox. ac.)

Epididymis sensitive to touch and while walking.

Offensive sweat on genitals (Calad., Merc., Sep, Sulph., Thuja; at night, Bell.).

Clinical.—Considered a good remedy to repress a too lively sexual desire, especially in children.

Concomitants.—Ill-humored, hates every one. Easily frightened at slight noises, after a nocturnal emission. The peculiar stool; its involuntary passage with flatus or urine. Fears to pass either less stool also escape.

